# Midflexion instability in primary total knee replacement: a review

**DOI:** 10.1051/sicotj/2015020

**Published:** 2015-08-05

**Authors:** Manjunath Ramappa

**Affiliations:** 1 James Cook University Hospital Middlesbrough TS4 3BW UK

**Keywords:** Knee, Midflexion instability

## Abstract

*Introduction*: Midflexion instability in primary total knee replacement (TKR) is an evolving concept. Successful treatment of instability requires an understanding of the different types of instability.

*Methods*: A literature review was performed to identify information pertinent to midflexion instability in primary total knee replacement, utilising PRISMA guidelines. Databases searched included Embase, Medline, All of the Cochrane Library, PubMed and cross references.

*Results*: Three factors, i.e., elevated joint line, multiradii femoral component and medial collateral ligament (MCL) laxity, were identified to influence midflexion instability. Literature suggested mediolateral instability at 30–60° of flexion as diagnostic of midflexion instability. Literature search also revealed paucity in clinical studies analysing midflexion instability. Most of the evidence was obtained from cadaveric studies for elevated joint line and MCL laxity. Clinical studies on multiradii femoral component were limited by their small study size and early followup period.

*Conclusion*: Elevated joint line, multiradii femoral component and MCL laxity have been suggested to cause midflexion laxity in primary TKR. Due to limitations in available evidence, this review was unable to raise the strength of overall evidence. Future well-designed clinical studies are essential to make definitive conclusions. This review serves as a baseline for future researchers and creates awareness for routine assessment of midflexion instability in primary total knee replacement.

## Introduction

Total knee replacement (TKR) procedures are currently on the rise. In England alone, a 10% increase in knee replacement procedures was reported in 2010 as compared to 2009 [1]. Recent National Joint Registry report [1] for England and Wales, suggested a revision TKR rate of 13.1% secondary to instability. Traditionally instability has been described as flexion or extension type. During knee arthroplasty surgery, extension and flexion instabilities are routinely assessed. In addition to these types of instabilities, midflexion instability has also been suggested to exist, more than a decade earlier [2]. However, unlike flexion or extension instability, midflexion instability in primary TKR has not been extensively studied. Hence, midflexion instability in primay TKR is still in the evolutionary phase. With an increasing number of primary TKR procedures, it becomes prudent to understand the various types of instability. Successful treatment of instability requires an adequate understanding of the different types of instability. Knowledge of factors contributing to instability is essential for both prevention and treatment purposes. Hence, a literature review was performed to identify the best available evidence pertinent to midflexion instability in primary total knee replacement.

## Methodology

**Figure 1.**
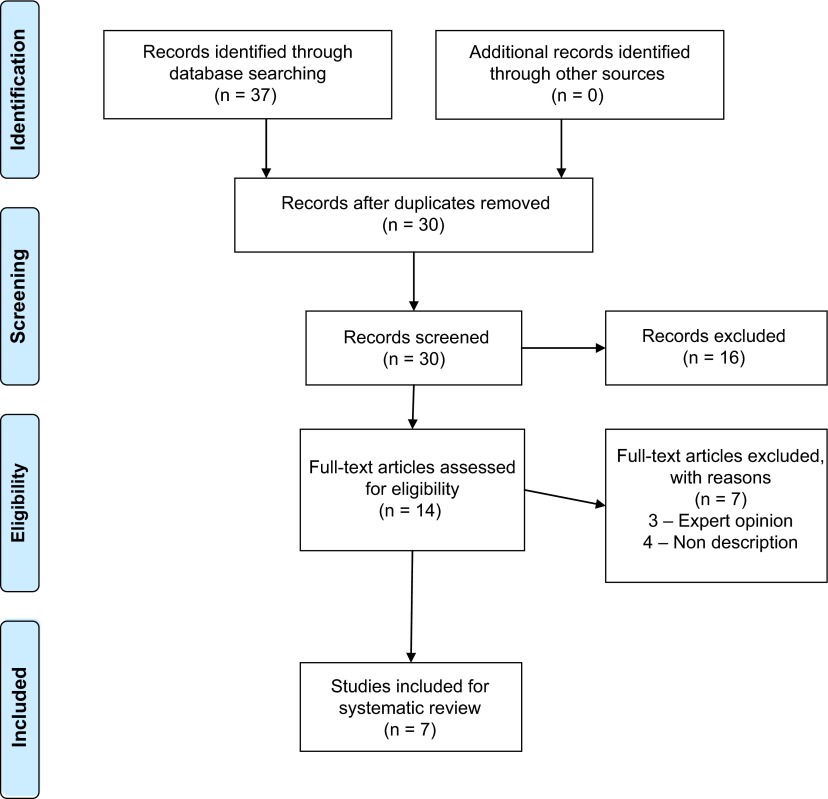
PRISMA 2009 flow diagram.

A systematic literature search was performed utilising Preferred Reporting Items for Systematic Reviews and Meta-Analyses (PRISMA) 2009 guidelines (Figure 1). Databases searched included Embase, Medline, All of the Cochrane Library, PubMed and cross references. Keywords utilised were knee, replacement, arthroplasty, instability, complications and revision. Boolean operators were also considered. Inclusion criteria were peer-reviewed published studies on midflexion instability in primary total knee replacement. Non-English literature was excluded. In the identification phase, the search was performed with keywords. In the screening phase, abstracts were reviewed for relevance. In the eligibility phase, full text was reviewed to assess for inclusion.

## Results

The literature search suggested that elevated joint line, multiradii femoral component and MCL laxity were associated with midflexion instability. Hence they were analysed further.

### Elevated joint line

Martin and Whiteside [2] in their cadaveric TKR study identified midflexion laxity in the coronal plane when the femoral component was shifted 5 mm proximally and anteriorly. Therefore joint line position was suggested to have a profound effect on midflexion instability. This could occur in the presence of well-balanced flexion and extension gaps. Elevation of the joint line alters the flexion-extension axis which can subsequently lead to laxity of the posterior capsule, PCL and collateral ligaments [2–4] at midflexion range i.e., 30–60° and thereby contribute to midflexion instability. Emodi et al. [5] in their cadaveric study showed a statistically significant increase in PCL strain with joint line elevation more than 2 mm in cruciate retaining TKR, thereby contributing to midflexion instability. Thus, joint line elevation provides little influence on cruciate sacrificing TKR systems. However, the PCL function is not the only factor influenced by joint line elevation. Joint line elevation also alters the distance between the attachment sites of the collaterals and the flexion axis. As the flexion axis goes higher, the distance between the collateral ligament attachment sites comes closer [6]. This effect is pronounced in midflexion and thereby contributes to midflexion instability. Elevated joint line also leads to other problems such as anterior knee pain and decreased flexion secondary to alteration in patella femoral mechanics, patella strain and alteration in patella position in relation to the joint axis [4]. Whether these factors also contribute to midflexion instability needs further investigation.

A large distal femoral cut to compensate for preoperative flexion contracture is a common reason for an elevated joint line in primary TKR and subsequent midflexion instability [3]. Hence, flexion contractures need to be addressed with alternative methods such as capsular release and osteophyte excision to prevent elevation of joint line. Distal femoral augments could also be considered in such situation to prevent joint line elevation [7]. In clinical practice, average joint line elevation in primary TKR has varied from 1 mm to 4.3 mm [8–11]. Snider and Macdonald [11] showed that joint line elevation more than 8 mm was associated with lower postoperative KSS scores. This group of patients also had lower preoperative scores. The overall difference in pre- and postoperative KSS scores was not affected by joint line elevation of 8 mm. Babazadeh et al. [7] in their randomised controlled trial comparing conventional TKR with computer-assisted TKR suggested that joint line depression of greater than 2 mm was associated with poor International Knee Society clinical scores at 2 years. However, it did not affect the quality of life. Figgie et al. [12] found better Mayo Clinic knee scores if the joint line was restored to within 8 mm of the preoperative position. Cruciate retaining and sacrificing TKR provided similar effect on the joint line [8, 11]. However, none of these clinical studies has reported midflexion instability. Therefore, further clinical studies analysing midflexion instability are essential. Currently, evidence correlating joint line elevation to midflexion instability is limited to cadaveric studies.

### Multiradius design

Wang et al. [13] performed a kinematic study to compare single radius and multiradii TKR designs. The study identified mediolateral instability in multiradii TKR design at midflexion range, which coincided with the flexion angle during transition between the different radii. This transition in axis occurs between 30 and 45° of flexion. However, this phenomenon has not been described in normal knees, which also have multiple radii. This study also compared midflexion movements between single radius and multiradii TKR designs during “stand to sit” motion, in patients with posterior stabilised knee. The study suggested increased abduction movement in the multiradii TKR group on lowering the body with the knee in the midflexion range, thereby contributing to midflexion instability. Also, there was higher knee extensor torque in the single radius TKR group for similar amount of quadriceps activation, as compared to multiradii TKR group. This was secondary to a more posterior position of flexion extension axis in the single radius group. A similar effect was noted by Wang et al. [14] when patients with bilateral TKRs, with single radius TKR in one leg and multiradii TKR in the other leg, were studied. Collateral isometry was also better maintained in the single radius group suggesting better midflexion stability. Kessler et al. [15] performed in-vivo fluoroscopic studies utilising finite helical axes and demonstrated increased spread for the flexion-extension axes across condyles during stair climbing in multiradii TKR. During stair climbing a more uniform movement was observed in single radius TKR. The study also showed increased varus-valgus laxity in the midflexion range in multiradii TKR. Thus the study suggested the possibility of midflexion instability with multiradii knee designs. However, all these studies were limited by the small numbers and short postoperative follow up.

Saleh et al. [16] suggested that the quadriceps could take more than 2 years, to regain preoperative levels of strength following TKR. Therefore in the long term, the difference in quadriceps activation between single and multiradius TKR designs may not be significant. Currently it is not clear if increased or diminished quadriceps activation causes midflexion instability. Also, in flexion, the anterior parts of collateral ligaments are taut whereas in extension the posterior parts of collateral ligaments are taut. At different positions of flexion and extension, different bundles within the collaterals become slack and taut [17]. Therefore, it seems logical to believe that multiradii TKR designs should be able to activate different collateral bundles throughout flexion and extension. Outcome studies [7, 18, 19] comparing single and multiradii TKR designs have not identified any midflexion instability. Also long-term studies on multiradii knee designs [20–22] with both cruciate retaining and sacrificing implants, have not suggested or identified any concerns regarding midflexion instability. However, nonidentification could have occurred due to under recognition of this phenomenon. Therefore, future studies have to analyse the effect of multiradii TKR designs on midflexion instability at long-term follow up.

### Medial collateral ligament (MCL) laxity

Medial soft tissue release is frequently performed to improve balancing in TKR. Inadvertent release can lead to iatrogenic MCL injury. Valgus knees [23] have a tendency for midflexion instability post-TKR, due to preexisting MCL laxity. Kinematic study [24] on cadaveric knees suggested that the superficial MCL behaved in a near-isometric fashion, however it was lax at the midflexion range of 30–50°. This effect was not noticed with LCL. Therefore any laxity in MCL will aggravate this situation leading to midflexion instability. This is especially seen if medial release includes the superficial MCL, especially the anterior part. To test isolated MCL function in clinical practice, the knee is tested in 30° of flexion. Similarly during TKR, an intraoperative assessment should consist of valgus and varus stress at midflexion. Cadaveric studies [25, 26] have suggested that superficial MCL release is associated with increased valgus laxity at flexion range 30–90° suggesting both midflexion and flexion laxity for both cruciate retaining and cruciate sacrificing knee replacements. However, this laxity was more pronounced in cruciate sacrificing TKR.

Treatment for MCL laxity will need to be considered in conjunction with flexion and extension assessment, location and extent of tear or laxity. Treatment options include non-operative, repair, augmentation, reconstruction or conversion to a constrained prosthesis [27–31]. Leopold et al. [31] showed good results with primary repair or reattachment with a nonconstrained prosthesis at two years, without any recurrent instability. Healy et al. [27] did not notice recurrent instability at four to nine year follow up, with medial reconstruction. PCL sacrifice has been suggested to increase instability and thereby needing a constrained implant in such situations with good results [29, 30]. However, currently there is no consensus regarding the best treatment option [30].

## Conclusion

Midflexion instability in primary TKR is an evolving concept. Elevated joint line, multiradii TKR and MCL laxity have been suggested to contribute to midflexion instability. Evidence for elevated joint line and MCL laxity was predominantly obtained from cadaveric studies. While evidence for multiradii knee was obtained from clinical studies, however due to the small study populations with short postoperative followup, their validity was limited. Midflexion instability has been diagnosed by assessing varus – valgus instability in midflexion range i.e., 30–60°, in all these studies. This examination should form part of routine intra- and postoperative assessment. The authors believe that paucity in the literature is secondary to under recognition of this process. Future well designed clinical studies are essential to confirm the findings from these studies. Due to the low quality of available evidence, it is difficult to make any definitive conclusions. Nonetheless, this review will form a baseline for future researchers. Arthroplasty surgeons need to be aware of this type of instability and routine TKR assessment should include midflexion knee examination.

## Conflict of interest

MR declares no conflict of interest in relation with this review.
